# Prevention of Nonalcoholic Hepatic Steatosis by Shenling Baizhu Powder: Involvement of Adiponectin-Induced Inhibition of Hepatic SREBP-1c

**DOI:** 10.1155/2020/9701285

**Published:** 2020-06-08

**Authors:** Kairui Tang, Yuanjun Deng, Chuiyang Zheng, Huan Nie, Maoxing Pan, Runsen Chen, Jiqian Xie, Qinhe Yang, Yupei Zhang

**Affiliations:** ^1^School of Traditional Chinese Medicine, Jinan University, Guangzhou 510632, China; ^2^Formula-Pattern Research Center School of Traditional Chinese Medicine, Jinan University, Guangzhou 510632, China; ^3^The First Affiliated Hospital of Jinan University, Guangzhou 510630, China

## Abstract

**Background:**

Nonalcoholic fatty liver disease (NAFLD) is a common chronic liver disease worldwide, and its incidence is increasing annually, but there is currently no specific drug for treating NAFLD. Shenling Baizhu powder (SL) is a safe herbal compound commonly used in clinical practice. Our previous research has shown that SL has the effect of preventing NAFLD, but its specific mechanism has not been determined. In this study, the potential mechanism of SL on NAFLD was explored by *in vivo* experiments.

**Methods:**

Wistar rats fed a choline-deficient amino acid-defined diet (CDAA) were treated with SL for 8 weeks. Then, serum samples were collected to obtain biochemical indicators; adipose tissue and liver samples were collected for pathological detection; a moorFLPI-2 blood flow imager was used to measure liver microcirculation blood flow, and a rat cytokine array was used to screen potential target proteins. The expression of liver adiponectin/SREBP-1c pathway-related proteins was determined by Western blotting.

**Results:**

SL effectively reduced the liver wet weight, as well as the levels of total cholesterol (TC) and triglyceride (TG) in the liver, and ameliorated liver injury in CDAA-fed rats. Pathological examinations showed that SL markedly reduced liver lipid droplets and improved liver lipid accumulation. In addition, the detection of liver blood flow showed that SL increased liver microcirculation in CDAA-fed rats. Through the cytokine array, a differentially expressed cytokine, namely, adiponectin, was screened in the liver. Western blotting assays showed that SL increased the expression of adiponectin and phosphoacetyl-CoA Carboxylase (p-ACC) in the liver and decreased the expression of steroid regulatory element-binding protein-1c (SREBP-1c) and fatty acid synthase (FAS).

**Conclusion:**

These results suggest that SL can increase the levels of adiponectin in the liver and serum and can inhibit the expression of SREBP-1c, thereby regulating systemic lipid metabolism and reducing liver lipid accumulation.

## 1. Introduction

Nonalcoholic fatty liver disease is a clinicopathological syndrome characterized by excessive lipid deposition in hepatocytes in the absence of alcohol and other causes of liver damage. Owing to overnutrition, NAFLD has become a common chronic liver disease worldwide, and how to prevent and treat this disease is a global public health problem that needs to be solved urgently [1]. NAFLD can have serious consequences for patients, and some patients with simple fatty liver disease can develop inflammation, liver fibrosis and, finally, liver cancer [2]. The pathogenesis of NAFLD and its progression is a complex process, and multiple factors combined with the genetic susceptibility of individuals contribute to the complexity of the pathogenesis of NAFLD [3]. Among these factors, the crosslinking between liver lipid metabolism and peripheral fat lipid metabolism may play an important role in the pathogenesis of NAFLD [4].

In contrast to traditional cognition, adipose tissue may be considered an “endocrine organ” that can secrete adipocytokines, such as leptin and adiponectin [5]. Adiponectin is a rich adipocyte secreting factor, and a large number of studies have confirmed that it has a wide range of biological activities, can improve the insulin sensitivity of the main insulin target tissues, and plays an important role in the regulation of energy metabolism [6]. Currently, low adiponectinemia has been identified as an independent risk factor for metabolic-related diseases, such as type 2 diabetes, coronary heart disease, and obesity [7, 8]. Studies have shown that adiponectin can activate anti-NAFLD by activating AMP-activated protein kinase (AMPK), a key kinase that regulates cellular energy homeostasis, via the AdipoR1-related pathway [9].

SREBP-1c is mainly expressed in hepatocytes and adipocytes and is an important nuclear transcription factor in animal fat metabolism [10]. Fat accumulation in the liver is associated with enhanced expression of lipogenic genes, such as acetyl-CoA carboxylase (ACC), FAS, and stearoyl-CoA desaturase 1 (SCD1), which is regulated by SREBP-1c [11]. In NAFLD animal models, SREBP-1c mRNA and its active nuclear protein form are increased, demonstrating that SREBP-1c overexpression leads to lipid accumulation in the liver [12]. Clearly, SREBP-1c may play an important role in the pathogenesis of fatty liver.

There is increasing evidence that NAFLD is a multisystem disease with complex pathogenic factors, and no simple and effective treatments are available. The main treatments for NAFLD include lifestyle intervention, medication, and weight loss surgery [13]. At present, Chinese herbal medicine plays a potentially effective role in the treatment of NAFLD, and the development of related Chinese herbal medicine has good prospects [14, 15]. Shenling Baizhu powder is a traditional Chinese medicine compound commonly used in Chinese clinical practice. SL has a history of clinical use for thousands of years and is a commonly used medicine for the treatment of the gastrointestinal system [16]. In a previous study, we found that SL can play a role in the treatment of NAFLD through antioxidative stress, anti-inflammatory and lipid-lowering activities, and regulation of gut microbiota [17–19]. However, the mechanisms underlying the role of SL in regulating lipids have not been elucidated. Therefore, in this study, we explored the molecular mechanism governing the action of SL against NAFLD from the perspective of the interaction between the liver and peripheral fat using a choline-deficient amino acid-defined diet (CDAA) induced NAFLD rat model.

## 2. Materials and Methods

### 2.1. Medicines and Diet

SL is composed of 10 kinds of Chinese herbal medicines (purchased from Jiangyin Tianjiang Pharmaceutical Co., Ltd., China), and we used liquid chromatography-mass spectrometry (LC-MS) technology to measure seven active ingredients in this batch of SL, including ginsenoside Rb1, ginsenoside Rc, pachymic acid, atractylolide I, atractylolide II, atractylolide III, and glycyrrhizic acid, as listed in Table 1. The polyene phosphatidylcholine capsules (PPC) used in the positive control group were produced by Sanofi (Beijing) Pharmaceutical Co., Ltd. The formulated granules and polyene phosphatidylcholine capsules were dissolved in distilled water, placed in sterile glass vials, and stored at -4°C. Solutions were heated to room temperature in a water bath before use. The choline-sufficient amino acid-defined diet (CSAA) and choline-deficient amino acid-defined diet feeds in this experiment are all purified standard feeds that meet the US AIN-93 standard (purchased from Trophic Animal Feed High-tech Co. Ltd., China), and a list of feed ingredients is shown in Table S1.

### 2.2. Animals and Groups

Male Wistar rats (6 weeks old, 200 ± 20 g) were obtained from Shandong Laboratory Animal Center (Jinan, China). The study was carried out at the Institute of Laboratory Animal Science, Jinan University (Guangzhou, China), and strictly followed the relevant experimental procedures (animal ethics number: 20170922165139). Animals were maintained at a controlled temperature (20 ± 2°C) and humidity (60 ± 10%) with a 12 h light/12 h dark cycle. After one week of adaptive feeding, the rats were randomly divided into four groups according to their weight: (I) CSAA group (CSAA, *n* = 10), which was fed choline sufficiency feeds for 8 weeks, (II) CDAA group (CDAA, *n* = 10), which was fed choline deficiency feeds for 8 weeks, (III) CDAA+SL group (CDAA+SL, *n* = 10), which was also administered SL (30 g/kg/d) while feeding choline deficiency feeds for 8 weeks, (IV) CDAA+PPC group (CDAA+PPC, *n* = 10), which was also administered PPC (120 mg/kg/d) while feeding choline deficiency feeds for 8 weeks. During the experiment, each group of animals was free to drink and eat. The food intake and body weights of each group of rats were measured weekly.

### 2.3. Sample Preparation and Liver Surface Microblood Flow Scan

After 12 h of fasting, all rats were anesthetized with intraperitoneal injection of 3% pentobarbital (0.1 ml/100 g body weight). After laparotomy, we first used the moorFLPI-2 blood flow imager (Moor Instruments, UK) to scan the surface of the liver for blood flow. The distance between the scan head and the liver surface was approximately 20 cm. The image acquisition rate was one frame per second in the normal resolution mode. The duration of recording was 1 min. Blood samples were collected from the abdominal aorta for blood biochemical analysis after the liver surface scan. Finally, the liver, perirenal fat, and testicular fat were collected, weighed, and stored at -80°C for further experiments.

### 2.4. Biochemical Analysis

A given weight of the liver sample was weighed into a centrifuge tube, absolute ethanol was added, the tissue was homogenized for 2 min using a tissue grinder (Qiagen, Germany), and the sample was centrifuged at 3000 × g for 10 min at 4°C. Finally, the liver tissue homogenate supernatant was collected. TC and TG levels in liver homogenate were detected using TC and TG kits (Nanjing Jiancheng Bioengineering Institute, China). Alanine aminotransferase (ALT), aspartate transaminase (AST), high-density lipoprotein cholesterol (HDL-C), low-density lipoprotein cholesterol (LDL-C), glucose, TC, and TG levels in serum were detected using an automatic biochemical analyzer (Hitachi, Ltd., Japan). Serum adiponectin levels in rats were detected using a rat adiponectin ELISA kit (Cusabio Technology LLC, China).

### 2.5. Histopathological Examination of Liver Tissue and Perirenal Fat

Liver tissue fragments approximately 0.5 cm from the edge of the left hepatic lobules were selected and fixed with 4% paraformaldehyde for 24 h and then embedded in paraffin. After the samples were sectioned, hematoxylin and eosin (HE) staining and Masson staining were performed. The fresh liver tissue was embedded with optimal cutting temperature compound, and the sample was made into 10 *μ*m slices by freezing microtome (Leica Biosystems, Germany) and then stained with Oil Red O kits (Nanjing Jiancheng Bioengineering Institute, China). Similarly, the adipose tissue around the left kidney was placed in 4% paraformaldehyde for 24 h and then sectioned for HE staining.

### 2.6. Rat Cytokine Array Analysis

First, liver protein samples of the CSAA, CDAA, CDAA+SL, and CDAA+PPC groups were prepared. Due to cost issues, protein samples from each group were made by mixing 10 liver samples from each group. This step was only employed for preliminary screening. These samples were tested using a rat cytokine array (RayBiotech, Norcross, GA, USA), including 67 cytokines. Briefly, 2 ml of blocking solution was added to the array membrane and incubated for 1 h at room temperature. The blocked membrane was then incubated with 150 *μ*l of protein sample in a 4°C refrigerator overnight. Thereafter, the membrane was washed three times with washing buffer for 10 min each time. Next, 80 *μ*l of the detection antibody cocktail was added to each well. Membranes were incubated at room temperature for 1 h. Membranes were washed once more as mentioned previously, and then 80 *μ*l of Cy3 equivalent dye-conjugated streptavidin was added to each well. The device was covered with aluminum foil to avoid exposure to light or was incubated in a dark room. Incubation was performed at room temperature for 1 h. Finally, fluorescence detection and data analysis were performed.

### 2.7. Western Blot Analysis

RIPA lysate containing phosphatase inhibitor (Beyotime Biotechnology Co., China) was added to a centrifuge tube containing a certain amount of liver tissue and homogenized. Tissue homogenates were centrifuged at 10,000 × g for 5 min at 4°C, and the supernatants were collected. The BCA protein quantitation assay (Beyotime Biotechnology Co., China) was used to measure the total protein content of the homogenate supernatant. Equal amounts of protein from each group were loaded onto 10% sodium dodecyl sulfate-polyacrylamide gel electrophoresis (SDS-PAGE) and then transferred to PVDF membranes (Millipore, Germany). Membranes were incubated with primary antibody overnight at 4°C, and the antibodies used in this study were adiponectin (1 : 1000, Cell Signaling Technology, USA), phospho-AMP-activated protein kinase (p-AMPK) (1 : 1000, Cell Signaling Technology, USA), AMPK (1 : 500, Cell Signaling Technology, USA), SREBP-1c (1 : 1000, Santa Cruz Biotechnology, USA), p-ACC (1 : 1000, Cell Signaling Technology, USA), ACC (1 : 1000, Cell Signaling Technology, USA), and FAS (1 : 1000, Cell Signaling Technology, USA). After being washed, membranes were subsequently incubated with horseradish peroxidase (HRP)-conjugated secondary antibody (Cell Signaling Technology, USA). Protein bands were visualized with the ChemiDoc™ Touch Imaging System (Bio-Rad Laboratories, Inc., USA).

### 2.8. Statistical Analysis

Data were presented as mean ± standard deviation (S.D.) and statistically analysed with SPSS 20.0 (IBM Corp., Armonk, NY, USA). In multiple group experiments with parametric data, the differences between groups were assessed using one-way ANOVA to determine overall significance followed by the Bonferroni multiple comparison test. In the comparison of the body weight of each group, two-way ANOVA with repeated measures showed significant differences.

## 3. Results

### 3.1. SL Improved the Body Parameters of NAFLD Rats

**Figure 1:**
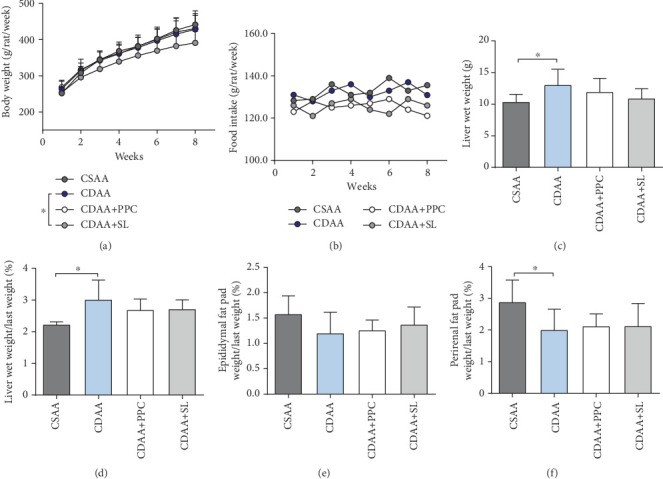
SL improved the body parameters of NAFLD rats. (a) Body weight growth curve of the four groups of rats. Two-way ANOVA with repeated measures showed significant differences. (b) The average weekly total food intake change for each rat. (c) Liver wet weight of the four groups of rats. (d) Liver wet weight/last weight (%) of the four groups of rats. (e) Epididymal fat pad weight/Last weight (%) of the four groups of rats. (f) Perirenal fat pad weight/last weight (%) of the four groups of rats. (c–f) use one-way ANOVA by Bonferroni as the statistical method. Data are presented as the mean ± S.D. for each group of rats (*n* = 10); ∗*P* < 0.05.

In this study, we continuously monitored weekly body weight changes and weekly total food intake for each group of rats (Figures 1(a) and 1(b). The body weight of each group increased gradually, and there was no significant difference in body weight between the CSAA group and the CDAA group. After SL intervention, body weight decreased compared with the CDAA group (*P* < 0.05). In terms of food intake, rats in each group maintained a relatively stable food intake, and there was no significant difference between the groups. After the rats were sacrificed, the liver wet weight, epididymal fat pad weight, and perirenal fat pad weight were measured to explore the correlation between the liver and visceral white fat. Compared with the CSAA group, the liver weight of the CDAA group significantly increased (*P* < 0.05). After SL or PPC intervention, compared with the CDAA group, the liver wet weight of rats tended to decrease, although the difference was not statistically significant (Figures 1(c) and 1(d)). When measuring visceral white fat, it was found that the weight of perirenal fat pads of rats fed with CDAA diet was significantly lower than that of CSAA group (*P* < 0.05), while the epididymal fat pads of the CDAA group also showed a tendency to decrease (although not statistically significant), suggesting that the choline deficiency diet-induced NAFLD rat model in this study may be related to these changes in visceral fat [20].

### 3.2. SL Improved Serum and Liver Biochemical Indicators

In this study, we examined the levels of ALT and AST in the serum of the abdominal aorta in each group, as shown in Table 2. The serum levels of ALT and AST in the CDAA group were significantly higher than those in the CSAA group (*P* < 0.01), suggesting that the CDAA diet can cause hepatocyte injury in rats; SL and PPC can reduce hepatocyte injury to varying degrees. In the serum lipid test, the levels of TC, TG, HDL-C, LDL-C, and glucose in the serum of rats fed the CDAA diet were not significantly different from those in the CSAA group, suggesting that the use of CDAA feed did not affect the lipids of rat serum. At the same time, there was no significant change in serum lipids in the CDAA+SL group compared with the CDAA group. In the detection of liver lipids, the levels of TC and TG in the livers of the CDAA group were significantly higher than those in the CSAA group. After SL or PPC intervention, the levels of TC and TG in the livers of the CDAA+SL group and CDAA+PPC group were significantly lower than those of the CDAA group, (*P* < 0.05, *P* < 0.01), suggesting that the livers of the CDAA group had TG accumulation. This result is consistent with the pathogenesis of NAFLD [21], and SL can improve lipid accumulation in the liver of rats.

### 3.3. Pathological Observation of Liver Tissue and Perirenal Fat Pad

**Figure 2:**
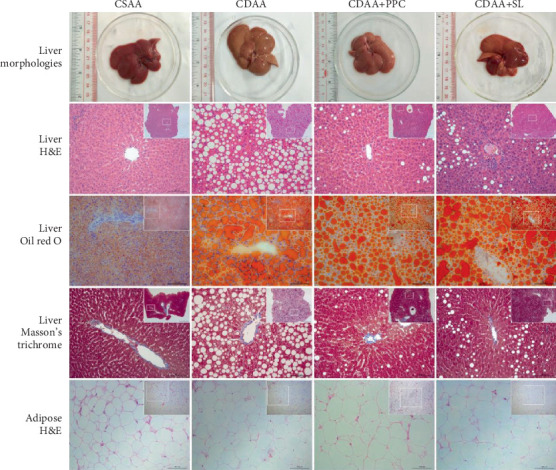
Pathological observation of liver tissue and perirenal fat pad. Representative images of macroscopic observation, hematoxylin-eosin staining, Oil Red O staining, and Masson's trichrome staining after 8 weeks in the respective groups. Among them, adipose tissue is the perirenal fat pad.

We attempted to reflect the pathological condition of NAFLD through different technical methods, as shown in Figure 2. In visual inspection of the entire liver, it was found that the liver volume of the CDAA group increased, the color was yellowish, and the capsule was tight when compared with the CSAA group. When SL or PPC was intervened, the liver volume was smaller than that of the CDAA group, and the color was biased to the CSAA group. The liver's H&E and Oil Red O staining revealed that the liver tissue of the CDAA group was scattered with a large number of orange-red lipid droplets of varying sizes, and a large number of lipid droplets squeezed the nucleus on one side of the cytoplasm. The arrangement of liver cells is disordered, the intracellular structure is unclear, and the hepatic lobular cord structure disappears; when SL or PPC intervention, the overall liver condition was significantly improved compared with the CDAA group. It can be seen from the steatosis score that the liver of the CDAA group has more severe steatosis than the CSAA group. SL intervention can improve the degree of liver steatosis (*P* < 0.05), as show in Figure S1 A. Masson staining can reflect the severity of fibrosis in liver tissue, but in this study, no significant fiber status was observed in the four groups, suggesting that the use of a CDAA diet for 8 weeks may not be sufficient for liver fibrosis. In the H&E staining of the perirenal fat pad, the adipocytes in the CDAA group exhibited a larger average volume compared to the CSAA group. At the same time, in the same observation area, the CDAA group had more adipocytes (*P* < 0.05), as show in Figure S1 B, C. After SL intervention, the number and average volume of adipocytes tended to improve compared with the CDAA group, although the trend was not obvious.

### 3.4. SL Improves Liver Perfusion in NAFLD Rats

**Figure 3:**
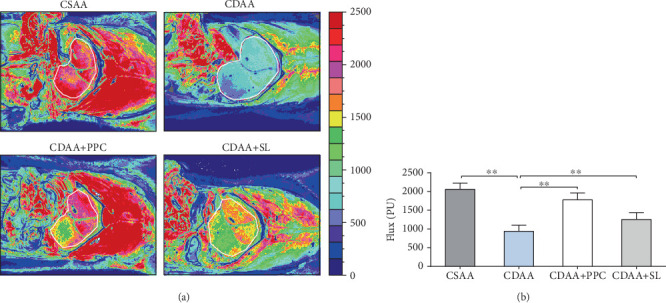
Liver perfusion alterations in the respective groups. (a) Representative images of liver perfusion in four groups of rats were evaluated by laser speckle flowmetry, with red areas corresponding to high perfusion and high flux areas and blue areas indicating low perfusion and low flux. (b) Scatter plots of liver microvascular blood flow in each group. We analyzed the average perfusion volume of the liver surface in the area within the white line in (a). One-way ANOVA by Bonferroni was used as the statistical method. Data are presented as the mean ± S.D. for each group of rats (*n* = 10); ^∗∗^*P* < 0.01.

The increase in liver microcirculatory disturbances could play a role in the pathogenesis of liver injury and are key components of NAFLD [22]. Therefore, in this study, we measured the blood flow on the liver surface of each group of rats. It can be visually observed from Figure 3 that the mean blood flow on the liver surface of the rats in the CDAA group was significantly lower than that in the CSAA group (*P* < 0.01), and the mean blood flow decreased by approximately 55%. The blood flow on the liver surface of the CDAA+SL group and the CDAA+PPC group was significantly improved compared with that of the CDAA group. The mean blood flow increased by approximately 34% and 90%, respectively.

### 3.5. Effect of SL on 67 Cytokines in Rat Liver

**Figure 4:**
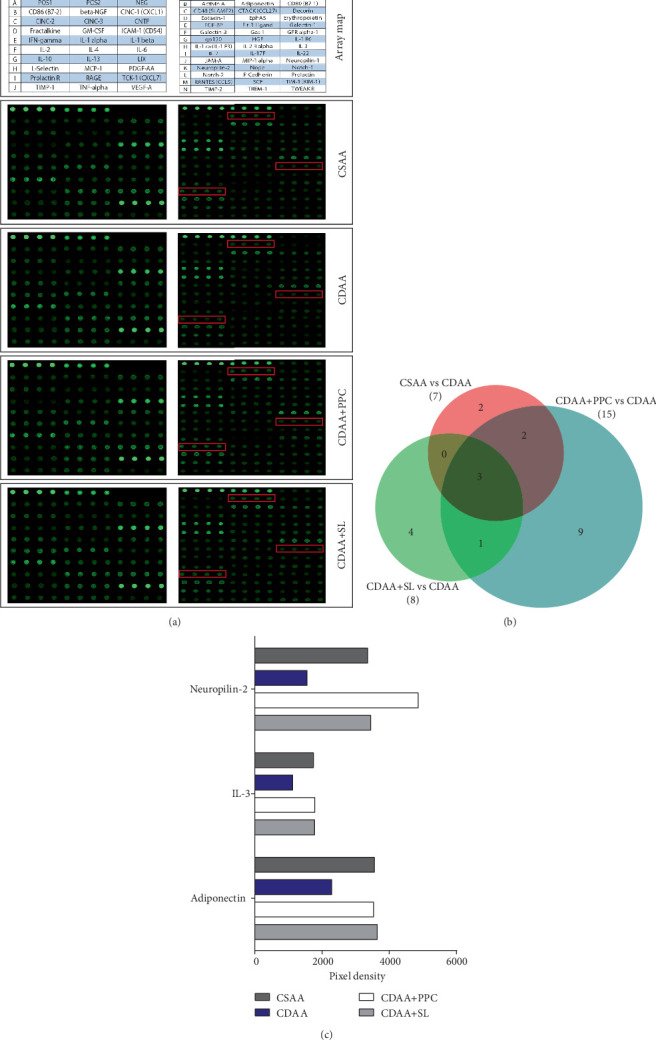
Effect of SL on 67 cytokines in rat liver. (a) The alignment of 67 cytokines on the rat cytokine antibody array. The arrays of neuropilin-2, IL-3, and adiponectin (with significant differences) are marked with small red boxes. (b) Venn diagram of differential expression of antibodies. (c) Bar graph shows the density of the array where neuropilin-2, IL-3, and adiponectin are located and the density of the spots normalized to the intensity of the positive control spots to allow for comparisons.

To determine the specific mechanism of SL in the prevention of NAFLD, we used the rat cytokine array kit to screen for differentially significant cytokines in liver homogenates, as shown in Figure 4(a). We used fold change ≤ 0.67 or fold change ≥ 1.5 and fluorescence signal > 150 as the screening conditions. In this study, a total of three cytokines were screened, namely, neuropilin-2, interleukin 3 (IL-3), and adiponectin, as shown in Figure 4(b). Compared with the CSAA group, neuropilin-2, IL-3, and adiponectin were all expressed at low levels in the CDAA group; when SL or PPC was employed, the levels of three cytokines in rat liver were significantly increased, suggesting that neuropilin-2, IL-3, and adiponectin may be the main target proteins for the efficacy of SL. In the following experiments, we mainly used adiponectin as the entry point for further verification.

### 3.6. SL Increases Adiponectin Levels in NAFLD Rats

**Figure 5:**
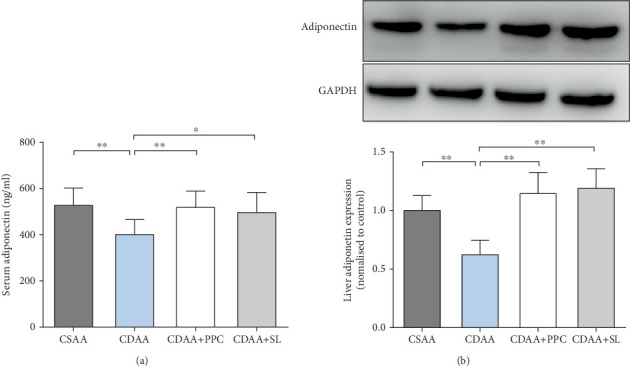
SL increases adiponectin levels in NAFLD rats. (a) Serum adiponectin levels in each group. (b) Representative Western blot and semiquantitative analysis of liver adiponectin in each group. One-way ANOVA by Bonferroni was used as the statistical analysis method. Data are presented as the mean ± S.D. for each group of rats (*n* = 10); ^∗^*P* < 0.05, ^∗∗^*P* < 0.01.

Adiponectin is the most abundant adipose-specific adipokine, and there is evidence that a decrease in adiponectin results in insulin resistance [23]. In this study, we used an enzyme-linked immunosorbent assay to detect the level of adiponectin in the serum of each group. From Figure 5(a), it was found that serum adiponectin levels were significantly decreased in the CDAA group compared with the CSAA group (*P* < 0.01); serum adiponectin levels were significantly increased after treatment with SL or PPC. Similarly, we used Western blotting to detect the level of adiponectin in the livers of rats. As shown in Figure 5(b), it can be clearly found that the adiponectin of the four groups had similar changes with serum adiponectin, suggesting that SL causes increased adiponectin content in NAFLD rats. Finally, AdipoR2 mRNA levels in the liver, which is the main receptor of adiponectin in the liver [24], were detected by RT-PCR. The results showed that the mRNA level of AdipoR2 in the CDAA group was lower than that in the CSAA group (*P* < 0.05), and SL intervention could increase the mRNA level of AdipoR2 in the rat liver (*P* < 0.05), as shown in Figure S2.

### 3.7. Effect of SL on the AMPK/SREBP-1c Pathway in the Livers of NAFLD Rats

**Figure 6:**
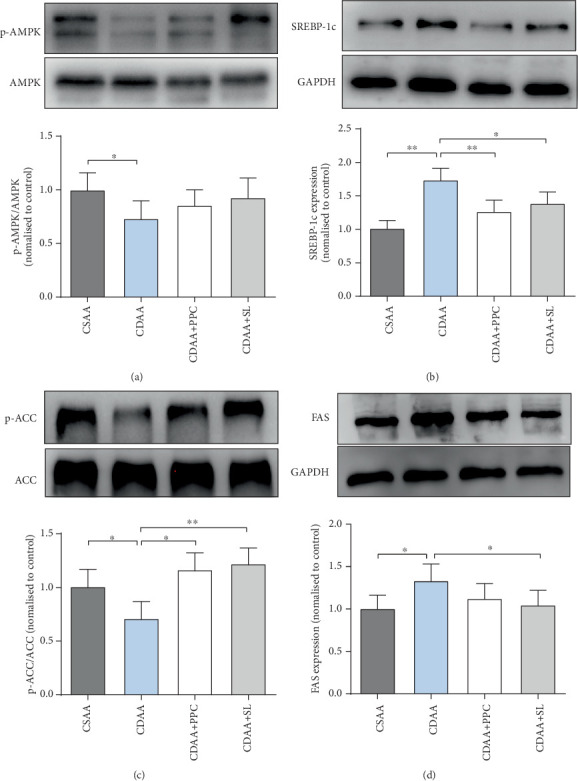
Western blot analysis of protein levels associated with the AMPK/SREBP-1c pathway in rat liver. (a) Representative Western blot and semiquantitative analysis of p-AMPK and AMPK protein in each group. (b) Representative Western blot and semiquantitative analysis of SREBP-1c protein in each group. (c) Representative Western blot and semiquantitative analysis of p-ACC and ACC protein in each group. (d) Representative Western blot and semiquantitative analysis of FAS protein in each group. One-way ANOVA by Bonferroni was used as the statistical method. Data are presented as the mean ± S.D. for each group of rats (*n* = 8 − 10); ^∗^*P* < 0.05, ^∗∗^*P* < 0.01.

Steroid regulatory element-binding protein-1c is an important nuclear transcription factor for lipid metabolism. It has been found that overexpression of SREBP-1c will disrupt lipid metabolism, resulting in excessive fat accumulation in nonadipose tissue [25, 26]. In this study, we examined the expression of SREBP-1c protein in the liver of each group of rats, as shown in Figure 6(b). Compared with the CSAA group, SREBP-1c was highly expressed in the liver of the CDAA group (*P* < 0.01). When SL or PPC was intervened, the expression of SREBP-1c protein was significantly decreased. Animal experiments showed that phosphorylation of AMPK inhibited the expression of SREBP-1c protein [27]. Therefore, we examined the relative expression of p-AMPK and AMPK in the livers of each group. The relative expression of p-AMPK in the CDAA group was significantly lower than that in the CSAA group (*P* < 0.05), while the relative expression of p-AMPK in the CDAA+SL group had an upward trend compared to the CDAA group. ACC and FAS are key enzymes in lipid metabolism synthesis and are downstream pathway proteins of SREBP-1c. From Figures 6(c) and 6(d), it can be observed that compared with the CDAA group, the expression of FAS protein in the liver of the CDAA+SL group was downregulated (*P* < 0.05) and the expression of p-ACC protein was upregulated (*P* < 0.01), suggesting that SL can reduce the activity of liver lipid synthesis in NAFLD rats established by choline deficiency.

## 4. Discussion

At the onset of disease, NAFLD has two important characteristics: hepatic triglyceride accumulation and insulin resistance. These properties emerge at the same time as increased lipotoxicity from high levels of free fatty acids, free cholesterol, and other lipid metabolites [28, 29]. Clinical evidence suggests that adiponectin levels are high in normal human serum; in contrast, adiponectin levels are observed to decrease in obese patients and those with hepatic steatosis [30]. Experimental evidence also shows that adiponectin and adiponectin receptor signaling may have a key role in improving nonalcoholic fatty liver disease [31]. According to current research, Shenling Baizhu powder has significant lipid-lowering effects, improves insulin resistance, and reduces the accumulation of triglycerides in the liver [32, 33]. To further reveal the mechanisms underlying the hepatocyte protective effects of SL, we used the CDAA diet to establish NAFLD rats, which is the most commonly used and reproducible nutritional model. The results from this study reveal that SL can increase adiponectin levels in NAFLD rats and decrease the protein expression of SREBP-c and rate-limiting enzymes in lipid metabolism pathways.

In this study, we observed that the use of the CDAA diet did not alter the changes in serum lipid and blood glucose levels in rats, but serum AST and ALT levels were significantly increased. SL or PPC can alleviate liver damage caused by the lack of choline, while it can significantly improve liver lipid accumulation. At the same time, it can be clearly observed from the pathological map that the pathological manifestations of NAFLD characterized by steatosis, lipid accumulation, and unclear hepatic lobules were observed in the liver of the CDAA group [13]. SL can significantly improve these characteristics. Notably, there were two points in the results that are slightly contradictory to our previous expectations. First, there were no significant changes in inflammatory factors (TNF-*α*, IL-1*β*, and IL-6) in the cytokine screening of liver tissue homogenates [34]. Second, no significant liver fibrosis was found in the liver in the pathology of Masson staining. These two findings suggest that this discrepancy may be related to the modeling time. The modeling time of 8 weeks was not enough to produce obvious liver fibrosis and liver inflammation.

According to recent reports, there may be many factors involved in the changes of liver blood flow, including the morphology and function of liver parenchymal cells and vascular endothelium, secreted products of Kupffer cells and stellate cells (such as nitric oxide, pro-fibrotic cytokines) [22]. Experimental research reports that adiponectin, which is closely related to the vascular system, can reduce endothelial cell apoptosis by activating AMPK and cyclooxygenase 2 (COX-2) pathways, and promote the production of nitric oxide, thereby improving atherosclerosis and increasing blood flow [35]. Aprahamian reported that the increased circulating adiponectin level can increase the vascular endothelial growth factor-A expression, vascularity, and blood flow perfusion in adipose tissue [36]. Moreover, clinical studies have shown that adiponectin plays an active role in the pathophysiology of vascular disease in patients with NAFLD [37]. Speckle blood flow imaging technology is a new auxiliary technique for evaluating liver function [38]. In this study, the liver perfusion of the CDAA group was significantly lower than that of the normal group, and SL increased the liver perfusion. At the same time, it was found that the changes of liver perfusion in each group were consistent with the changes in the level of serum adiponectin. In summary, our finding may be partially explained by the possibility that the increased liver perfusion by SL may be associated with the increased adiponectin level. However, further experiments are needed to clarify the exact mechanism.

The communication between adipose tissue and liver is crucial to the maintenance of systemic energy homeostasis [39]. Free fatty acids that originate from lipolysis of triglycerides in adipose tissue are delivered through blood to the liver [40]. When the disposal of fatty acids through beta-oxidation or the formation of triglycerides is overwhelmed, the liver will have lipid accumulation [41]. In this study, rats fed a CDAA diet had lower perirenal fat and testicular fat weights than rats in the CSAA group, and the morphology of perirenal adipocytes was different from that of the control group. Overall, these findings suggest that lipid redistribution is closely related to the pathogenesis of NAFLD. In fact, polypeptides secreted by adipocytes, termed adipokines, play an important role in systemic energy homeostasis [42]. In the screening of 67 cytokines in liver homogenate, we found marked differences in the adiponectin level among the groups, which were further confirmed by western blotting. Adiponectin is an important regulator in the regulatory network of lipid metabolism, and blood glucose homeostasis in the body and is considered a potential target for the treatment of NAFLD [43–45]. Adiponectin has repeatedly been shown to enhance insulin sensitivity [46]. Therefore, we hypothesize that adiponectin secreted by adipocytes may be strongly associated with the therapeutic effects of SL. We used enzyme-linked immunosorbent assay and Western blotting to detect the adiponectin level in rat serum. The adiponectin level in the CDAA group was significantly lower than that in the CSAA group, and adiponectin was significantly increased in rats after SL treatment.

Studies have shown that activating adiponectin-related signaling pathways inhibits the expression of SREBP-1c in the liver and reduces the expression of genes involved in liver adipogenesis and cholesterol synthesis [47, 48]. SREBP-1c is the main transcription factor that regulates hepatic *de novo* lipogenesis by insulin [49]. A clinical study showed that SREBP-1c in the liver of patients with NAFLD is highly expressed and activated [50]. The experimental results of this study showed that the protein expression of SREBP-1c in the CDAA group was significantly higher than that in the CSAA group. In contrast, SREBP-1c in the liver of the CDAA+SL group showed low activation status, and SL significantly reduced its expression. The phosphorylation of ACC decreases the synthesis of malonyl CoA and ultimately stimulates fatty acid oxidation [51]. Fatty acid synthase can catalyze the de novo synthesis of liver fatty acids and increase fat content [52]. From the results of Western blotting in this study, SL may regulate liver lipid metabolism through phosphorylation inhibition of acetyl-CoA carboxylase and downregulation of fatty acid synthase expression.

## 5. Conclusions

Taken together, the results of our study reveal a role for SL in the treatment of NAFLD. SL may target adiponectin as an intermediate communication bridge to adjust the lipid distribution and metabolism of liver and peripheral adipose tissue. Then, adiponectin regulates the expression of SRBEP lipid metabolism pathway-related proteins in the liver to exert anti-NAFLD effects. However, future research is warranted to determine how SL regulates the secretion of adiponectin from peripheral adipose tissue.

## Figures and Tables

**Table 1:** Composition information of each Chinese herbal medicine in SL.

Chinese name	Latin name	Composition ratio	Active ingredient (concentration *μ*g/L)
Ren Shen	Ginseng Radix et Rhizoma	5	Ginsenoside Rb1 (95.89 *μ*g/L) Ginsenoside Rc (146.00 *μ*g/L)
Fu Ling	Poria	5	Pachymic acid (5 *μ*g/L)
Bai Zhu	Atractylodis Macrocephalae Rhizoma	5	Atractylolide I (10.38 *μ*g/L) Atractylolide II (5 *μ*g/L) Atractylolide III (11.14 *μ*g/L)
Shan Yao	Dioscoreae Rhizoma	5	
Bai Bian Dou	Lablab Semen Album	4	
Lian Zi	Nelumbinis Semen	3	
Gao cao	Glycyrrhizae Radix et Rhizoma Praeparata Cum Melle	3	Glycyrrhizic acid (372.88 *μ*g/L)
Yi Yi Ren	Coicis Semen	3	
Jie Geng	Platycodonis Radix	2	
Sha Ren	Amomi Fructus	2	

**Table 2:** Serum and liver biochemistry.

Items	CSAA	CDAA	CDAA+PPC	CDAA+SL
Serum parameters				
ALT (U/L)	26.0 ± 6.79	61.6 ± 32.08^##^	40.60 ± 14.71	46.20 ± 15.24
AST (U/L)	69.00 ± 19.06	151.56 ± 45.51^##^	105.25 ± 20.01^∗^	106.25 ± 17.19^∗^
TC (mmol/L)	1.08 ± 0.24	1.02 ± 0.34	1.37 ± 0.26	1.18 ± 0.15
TG (mmol/L)	0.34 ± 0.15	0.42 ± 0.47	0.50 ± 0.39	0.49 ± 0.28
HDL-C (mmol/L)	0.29 ± 0.09	0.31 ± 0.12	0.40 ± 0.07	0.35 ± 0.05
LDL-C (mmol/L)	0.17 ± 0.05	0.15 ± 0.03	0.24 ± 0.05∗∗	0.19 ± 0.04
Glucose (mmol/L)	7.62 ± 1.73	8.94 ± 1.77	8.16 ± 0.59	8.89 ± 0.82
Liver parameters				
TC (mmol/kgprot)	36.10 ± 8.45	56.34 ± 14.51^#^	30.53 ± 6.56^∗∗^	31.76 ± 5.65^∗∗^
TG (mmol/kgprot)	77.21 ± 26.97	171.33 ± 76.25^#^	74.74 ± 19.18^∗^	73.14 ± 32.03^∗^

^#^ *P* < 0.05, ^##^*P* < 0.01 vs CSAA; ^∗^*P* < 0.05, ^∗∗^*P* < 0.01 vs CDAA. *n* = 9 − 10.

## Data Availability

The data used to support the findings of this study are included within the article.

## Supplementary Materials

Supplementary Materials Supplementary materials include materials and methods, table and figures.
