# Supplementary material for: Prevention of Nonalcoholic Hepatic Steatosis by Shenling Baizhu Powder: Involvement of Adiponectin-Induced Inhibition of Hepatic SREBP-1c
Source: Oxid Med Cell Longev. 2020 Jun 8;2020:9701285. doi: 10.1155/2020/9701285 (PMC7533788; doi:10.1155/2020/9701285)
Supplement: Supplementary Materials — include materials and methods, table and figures. [file 9701285.f1.doc]

**Table of Contents**

Supplementary Materials and Methods..........................................................................1

Supplementary Table S1................................................................................................1

Supplementary Figure S1-2............................................................................................2

**Supplementary Materials and Methods**

**Real-time PCR**

Total RNA was extracted from the liver tissues of rats using Trizol reagent (Takara Biomedical Technology, Japan). AdipoR2 were reverse transcribed using the PrimeScript RT reagent Kit with gDNA Eraser (Takara Biomedical Technology, Japan) and quantified via TB GreenTM Premix Ex TaqTM Ⅱ (Takara Biomedical Technology, Japan). The following primers were purchased from Generay, Shanghai, China. Rats AdipoR2 (Sence: 5´GAAGGAGGGTCAACTCACCA3´; Antisence: 5´CATCAAGTTGGTGCCCTTTT3´). Rats GAPDH (Sence: 5´TCTCTGCTCCTCCCTGTTC3´; Antisence: 5´ACACCGACCTTCACCATCT3´).

**Supplementary Table S1**

Table S1: Nutrient composition of the feed used in the experiment.

| **Ingredient(g)** | **CSAA** | **CDAA** |
| --- | --- | --- |
| Amino Acid Mixture | 144 | 144 |
| Corn Starch | 100 | 100 |
| Dextrin | 100 | 100 |
| Sucrose | 404.2 | 406.7 |
| Corn Oil | 150 | 150 |
| Cellulose | 50 | 50 |
| Mineral Mixture | 39.3 | 39.3 |
| Vitamin Mixture | 10 | 10 |
| Choline Chloride | 2.5 | - |
| Total | 1000 | 1000 |

**Supplementary Figure S1-2**


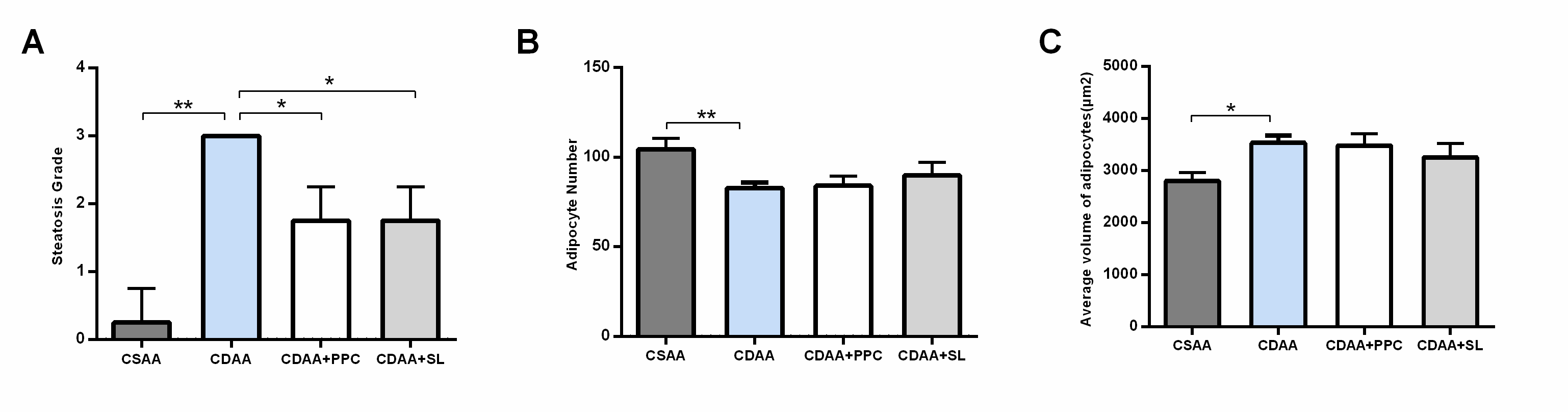


Figure S1: Relevant quantitative data in pathological observation. (A) The degree of liver steatosis in each group. Quantitative analysis of liver fatty degeneration of each group according to NAFLD activity score: 0 points (<5%); 1 point (5% -33%); 2 points (34% -66%); 3 points (> 66%). (B). Number of adipocytes in the perirenal fat pad. Count the number of cells in each group under the same area (200x). (C) Average volume of adipocytes in each group. One-way ANOVA by Bonferroni was used as the statistical method. Data are presented as the mean ± S.D. for each group of rats(n=3); *P<0.05, **P< 0.01.


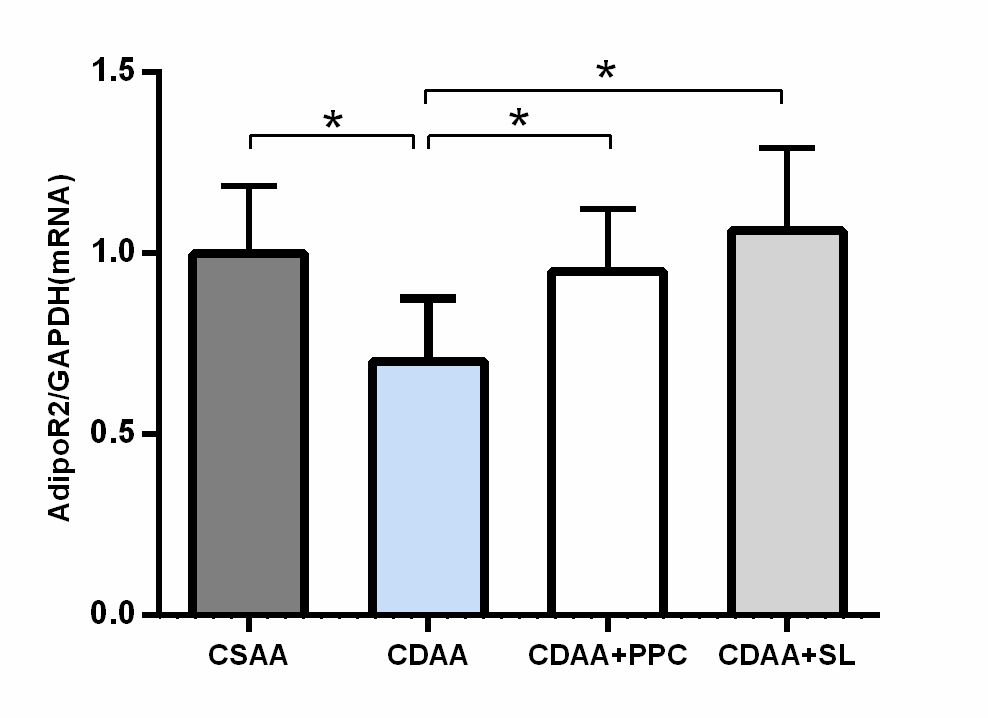


Figure S2: Expression of AdipoR2 in the liver of rats in each group. Hepatic mRNA levels of AdipoR2, as detected by real-time PCR. One-way ANOVA by Bonferroni was used as the statistical method. Data are presented as the mean ± S.D. for each group of rats(n=10); *P<0.05.
